# GORouter: an RDF model for providing semantic query and inference services for Gene Ontology and its associations

**DOI:** 10.1186/1471-2105-9-S1-S6

**Published:** 2008-02-13

**Authors:** Qingwei Xu, Yixiang Shi, Qiang Lu, Guoqing Zhang, Qingming Luo, Yixue Li

**Affiliations:** 1 The Key Laboratory of Biomedical Photonics of the Ministry of Education, HUST, Wuhan 430074, China; 2 Shanghai Center for Bioinformation Technology, Shanghai 200235, China; 3 Bioinformatics Center, Key Lab of Systems Biology, Shanghai Institutes for Biological Sciences, Chinese Academy of Sciences, Shanghai 200031, China

## Abstract

**Background:**

The most renowned biological ontology, Gene Ontology (GO) is widely used for annotations of genes and gene products of different organisms. However, there are shortcomings in the Resource Description Framework (RDF) data file provided by the GO consortium: 1) Lack of sufficient semantic relationships between pairs of terms coming from the three independent GO sub-ontologies, that limit the power to provide complex semantic queries and inference services based on it. 2) The term-centric view of GO annotation data and the fact that all information is stored in a single file. This makes attempts to retrieve GO annotations based on big volume datasets unmanageable. 3) No support of GOSlim.

**Results:**

We propose a RDF model, *GORouter*, which encodes heterogeneous original data in a uniform RDF format, creates additional ontology mappings between GO terms, and introduces a set of inference rulebases. Furthermore, we use the Oracle Network Data Model (NDM) as the native RDF data repository and the table function RDF_MATCH to seamlessly combine the result of RDF queries with traditional relational data. As a result, the scale of *GORouter *is minimized; information not directly involved in semantic inference is put into relational tables.

**Conclusion:**

Our work demonstrates how to use multiple semantic web tools and techniques to provide a mixture of semantic query and inference solutions of GO and its associations. *GORouter *is licensed under Apache License Version 2.0, and is accessible via the website: .

## Background

The currently preferred tool for uniform data presentation in systems biology, the syntactic and document orientated eXtensible Markup Language (XML), cannot satisfy the requirements of highly dynamic and integrated bioinformatics applications. However, Semantic Web [1] provides a universal mechanism for information exchange by describing, in a machine-interpretable way, the content of resources on the Web. The growing need for integration of diverse and heterogeneous data sets from distinct communities of scientists in separate biological research fields has thus been the major driving force to migrate from traditional XML to Semantic Web [2].

Gene Ontology [3] (GO, ) is by far the most widely used bio-ontology. As of August 2007, it contains approximately 23,700 terms, linked to a database of more than 16 million annotations of genes and gene products, originating from about 20 organisms. As a Semantic Web application domain, Gene Ontology Consortium provides a RDF-XML data file . It is an export of the database, containing both the GO vocabulary and associations between GO terms and gene products. However, this file has drawbacks, making it unsuitable for providing complex semantic query and inference services.

The first drawback is the lack of relationships between concepts among different GO subontologies, limiting the power of inference based on them. GO has three independent subontologies, Cellular Component, Biological Process and Molecular Function. The terms in the subontologies are structured as Directed Acyclic Graphs (DAG), and may have one or more parents with two types of relationships: '*is-a*' is a simple class-subclass relationship, while '*part-of*' represents a complex part-whole relationship. However, neither of them reflects the biological relationships among various subontologies. Several approaches, Lexical [4-8] and non-lexical [9-12], have been used to tackle this issue.

Lexical approaches are based on the fact that GO terms and definitions are themselves a type of semi-structured natural language. About 65% of all GO terms contain another GO term as a proper substring [4]. For example, the MF_*mannosyltransferase activity *_(GO:0000030) shares a substring with the CC_*mannosyltransferase complex *_(GO:0031501). The Obol project proposed a formal language to provide computable definitions that serve to differentiate a term from other similar terms [5]. Furthermore, Bada et al. designed 31 patterns to match term substrings to concepts and predicted an initial set of over 4000 associations [6]. Lexical methods mainly focus on the analysis of the compositional nature of Ontology terms, which leads to an increase in the number of relationships. The same ideas also could be applied to identify the dependence among various domains of biological knowledge, such as the Open Biological Ontology (OBO) family, chemical entity (ChEBI), BRENDA Tissue ontologies, and so on [8].

Statistical approaches based on the assumption that since some pairs of terms coming from different GO subontologies are annotated to the same gene or gene product, the relationships should reflect an actual interdependence between them. By analyzing the statistics of co-occurrence of GO terms in the model organism annotation databases of the Gene Ontology Annotation (GOA), Bada et al. developed the Gene Ontology Annotation Tool (GOAT) [9]. GOAT assists the Gene Ontology Next Generation (GONG) project [11] to convert GO Terms into a description-logic-based ontology (DAML+OIL). Similarly, Kumar mined the TIGR database to establish the corresponding patterns of association between terms in GO [10]. Other non-lexical methods, such as computing similarity in vector space, association rule mining, ontologies analysis, have also be introduced to address this problem [12].

The second drawback is that the RDF-XML data file is organized with a term-centric view of GO annotation data. All information is stored in a single file. The loading, querying and visualizing of massive amounts of RDF datasets are the main bottleneck of semantic web prototype applications [13]. Several semantic web tools, Sesame [14], Kowari [15], Jena2 [16], 3Store and RDFStore, have been developed and made available. Unfortunately, these repositories are not suitable for work with large amounts of data .

On the other hand, the scale of semantic web datasets of the life sciences increases dramatically. Many communities, such as GO, UniProt, UMLS, OMIM, KEGG and MGED, have provided download services for data encoded in RDF or Web Ontology Language (OWL) format. Correspondingly, semantic web prototype tools have been developed to address life science and health care requirements. For example, BioDASH [17] provides a *Drug Development Dashboard *that associates disease, compounds, drug progression stages, molecular biology, and pathways for a group of users. The YeastHub [18] and Bio2RDF [19] projects explore how the needs for data integration can be addressed by the semantic web and how a life sciences data warehouse can be built. However, most of the semantic web prototype applications create an RDF repository using the computers' main memory to speed up performance. This solution poses a high demand on the application server and is unable to satisfy the need for rapid growth of semantic web applications.

The third drawback is the lack of support for GOSlim. GOSlims are cut-down versions of the GO ontologies containing a subset of all terms in GO. They are particularly useful for giving a summary of the results of GO annotations of genomes, microarrays or cDNA collections [20,21]. However, GOSlim properties are not considered in RDF-XML data files.

## Results

In this paper, we present a RDF model *GORouter*, which mainly demonstrates how to use multiple semantic web tools and techniques to integrate heterogeneous resources and to create additional semantic relationships between different RDF datasets.

By introducing GLUE system [22] to create ontology mappings between pairs of terms coming from the three independent GO sub-ontologies, introducing a set of inference rulebases, and using the Oracle Network Data Model (NDM) [23] as the native RDF data repository, we believe that *GORouter *has the capability to allow complex semantic queries and inference services for GO and its associations.

### Datasets and software availability

*GORouter *is licensed under Apache License Version 2.0 and available for free download from the SourceForge website . Based on *GORouter*, we provide an application  for searching and browsing GO and its associations, and which also delivers additional functions such as semantic inference services.

## Discussion and conclusion

### Algorithm advance

In this section, we discuss some shortcomings of current algorithms for ontology mapping.

Firstly, finding associations using non-lexical and lexical approaches has little overlap [12]. Myhre et al. attempt multiple strategies to bridge this gap [24]. The GLUE system supports multiple learning strategies to generate *join probability distribution*. However, our project currently only employs an *annotation statistics *strategy. Integrating lexical learning strategies into the project will be the main focus of the next development phase.

Secondly, the GLUE system can currently not handle more sophisticated mappings (i.e. non one-to-one mapping) between GO terms. As an extended version of the GLUE system, CGLUE [25] can be used to exploit complex mappings.

Thirdly, the GLUE system only focuses on finding correspondences among the taxonomies of two given ontologies. Ontology specifies a conceptualization of a domain in terms of concepts, attributes and relations. The concepts provide model entities of interest in the domain, and they are typically organized into a taxonomy tree. Despite taxonomies being central components of ontologies, attributes and relations also need to be considered during the process of exploit mapping.

### RDF to OWL

OWL builds on RDF and adds more vocabulary along with formal computational definitions for reasoning. Compared with RDF, OWL facilitates greater machine interpretability of Web content. The OWL format is becoming the next generation of bio-ontology representation [26-29]. Several ontology editors, such as OBO-Edit [30], Protégé-OWL [31] and COBrA [32], can be used to perform the translation and provide Description Logic reasoning.

We currently use Oracle 10gR2 NDM as RDF repository, which does not incorporate native OWL support. The next generation, Oracle Spatial 11g, will support both RDF and OWL data management [33]. It is another important task for us to migrate *GORouter *from RDF to OWL format.

### Refinement and extension

The GO project is a collaborative effort to address the need for consistent descriptions of gene products in various databases. However, some molecular functions, biological processes and cellular components are not common to all life forms. GO uses the designator *sensu*, 'in the sense of', to name those species-specific terms. For instance, BP_*invasive growth (sensu Saccharomyces) *_(GO:0001403) represents the invasive growth process of Saccharomyces cell, which can only be used to annotate genes and gene products of the Saccharomyces Genome Database (SGD). These species-specific terms violate the species-independent principle of the GO vocabulary.

From another point of view, one could call this phenomenon a semantically-weak problem: the GO vocabulary has no control over the semantic context of term names. We will address this problem by introducing the NCBI organism classification (TAXON) into *GORouter*. By separating species-specific terms from the GO vocabulary, we plan to create a set of special GO subsets, which can be applied to the specified class of organism. Furthermore, the TAXON vocabulary can also be used to identify the species encoding gene products. By introducing TAXON, we can create richer relations across various GOs and their annotations.

Similarly, we also plan to introduce Sequence Ontology [34] (SO), a sister project of GO, to describe features and attributes of gene sequences and gene products. In recent years, the development of bio-ontologies has been very rapid [35,36]. As an essential part of OBO collection, GO development principles have been extended to many other biological domains and give an opportunity to introduce more ontology and annotations into *GORouter *to enrich the content of semantic relationships.

Gene Ontology is itself dynamic [37]. The development of GO terms and annotations reflects the current status of biological knowledge. For instance, the GO consortium has partially completed the subsumption hierarchy (a set of high-level terms) for the cellular component ontology, and the project is expected to be completed in 2007. The Plant-Associated Microbe Gene Ontology (PAMGO, ) Interest Group introduced a new set of terms representing pathogenic and symbiotic processes. Alongside the continuous improvement of GO ontology content, increasing model organism databases and genome annotation groups contribute annotation sets using GO terms.

In summary, all these changes indicate that the content of *GORouter *needs to be correspondingly augmented, refined and reorganized. These requirements provide two challenges: one is to improve model flexibility and the other is to adapt performance to the continual increase in size. By using multiple semantic web technologies and tools, we believe that *GORouter *can overcome these problems.

## Methods

### Metadata and data

Most of the original files come from the Gene Ontology Consortium, including MySQL relational data, the OBO format data of GOSlim, tab-delimited annotation files, and RDF XML format data with or without annotation. We encoded these heterogeneous resources in uniform RDF format, and created a set of RDF datasets (Reference YeastHub project). Each dataset consists of two RDF files, *metadata *and *data*.

**Figure 1:**
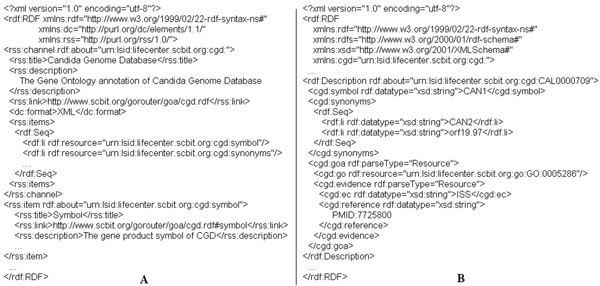
**A metadata and data RDF file of the Candida Genome Database (CGD) annotation dataset**. (A) The CGD *metadata *RDF file is encoded with RSS1.0, which can be easily shared by various biological research domains. (B) There is a CGD data RDF file associated with (A). We assign a unique LSID to each URL.

In order to increase the usability and portability, *metadata *RDF files (Figure 1A) are encoded with RSS1.0 (Rich Site Summary, ), including standard properties coming from the Dublin Core Metadata (DCM) vocabulary . Each resource of *metadata *is known as a channel and its contents as a 'RSS feed'. RSS applications can access these RSS-enabled sites and collect their feeds, therefore, these properties can be easily shared by various biological research domains. In *metadata *RDF files, we provided all standard definitions of properties as follows:

(1) ***Symbol***: is a standard gene product symbol.

(2) ***Synonyms***: a RDF sequence container for storing the synonyms of genes and gene products.

(3) ***GOA***: is a RDF omitting blank node with two sub-property elements: *go *and *evidence*, which indicates the GO Annotation. A gene product may have more than one annotation.

(4) ***GO***: is a LSID, which refers to an accession number of GO term.

(5) ***Evidence***: is a RDF omitting blank node with two sub-property elements: *ec *and *reference*, which refers to the evidence supporting the annotation. For a given annotation, more than one evidence may be associated with it. In *GORouter*, we only focus on credible evidence, such as Inferred by Curator (IC), Inferred from Direct Assay (IDA), Traceable Author Statement (TAS), and so on.

(6) ***EC***: indicates the evidence code for the annotation.

(7) ***Reference***: is a reference cited to support the annotation.

Each *metadata *RDF file has a *data *RDF file (Figure 1B) associated with it. We assign only one unique Life Science Identifier [38] (LSID) to each URL of *data *RDF files. Currently, only few databases provide LSIDs for their data. Therefore, we decided to assign these identifiers ourselves. Each LSID consists of up to five parts (URN:LSID:Authority:Namespace:Object: [Revision-ID]), in which URN:LSID is a mandatory prefix; Authority is the Internet domain of the organization which assigns the LSID to the resource; Namespace constrains the scope of the object; Object is an alpha-numeric describing the object; Revision-ID is the optional version of the object. For an example, there is a CGD (Candida Genome Database) gene whose database accession number is 'CAL0000849'. Thus, the LSID will be written as: 'urn:lsid:lifecenter.scbit.org:cgd:CAL0000849:1' or as a simpler style: 'urn:lsid:lifecenter.scbit.org:cgd:CAL0000849'.

### Ontology mapping

Given two ontologies *O*_1 _and *O*_2_, for each term A (*A *∈ *O*_1_), the ontology mapping algorithms attempt to find the most similar term B (*B *∈ *O*_2_). We describe this mapping as "A *mapping-to *B". Nowadays, there are over 23,700 GO terms, including approximately 7,800 Molecular Function terms, 2,000 Cellular Component terms and 13,900 Biological Process terms. Manual GO subontology mapping is not reliable, and it is therefore crucial to use algorithms and computational tools to assist experts to generate these mappings.

**Figure 2:**
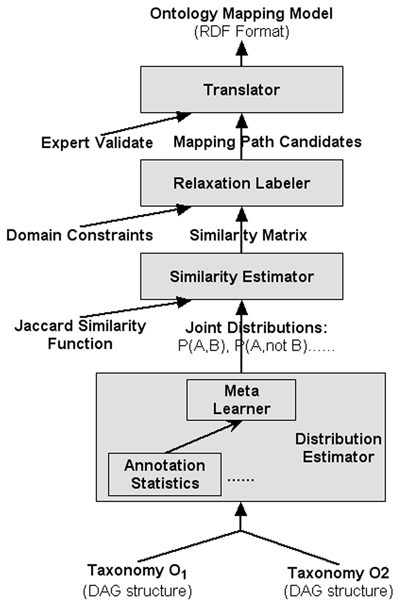
**GLUE System Architecture**. There are four modules are included in the GLUE system. The *Distribution Estimator *module uses multiple machine learning strategies to generate a *join probability distribution P*(*A*, *B*). The *Similarity Estimator *module uses the *Jaccard Similarity *function to construct a *similarity matrix*. The *Relaxation Labeler *module uses domain constraints and heuristic knowledge to improve the match accuracy. Finally, after validation by experts, the *Translator *module encodes the mapping paths with uniform RDF format and loads them into *GORouter*.

In this paper, we apply the GLUE system (as shown in Figure 2) to semi-automatically generate 6 types of mapping paths and translate them into a set of *GORouter Mapping Datasets*.

The core issue of mapping algorithms is how to measure the similarity between two terms. The GLUE system is based on the observation that many practical measures of similarity can be defined based solely on the *join probability distribution *of the terms involved. In *the Similarity Estimator *module, we use *the Jaccard Similarity *function (Formula 1) to calculate a similarity measure for any pair of terms coming from different GO subontologies.

$$Jaccard-sim(A,B)=P(A\cap B)/P(A\cup B)=\frac{P(A,B)}{P(A,B)+P(\bar{A},B)+P(A,\bar{B})}$$

The value of *P*(*A*, *B*) can be computed as the fraction of the instance universe that belongs to both A and B. In general, we cannot compute this fraction, because we do not know every instance in the universe. Hence, we estimate *P*(*A*, *B*) based on the data we have, namely, the GO annotations. We denote by *U*_*i *_the set of annotations given for GO subontology *O*_*i*_, by *N*(*U*_*i*_) the size of *U*_*i *_and by $N(U_{i}^{A,B})$ the number of annotations in *U*_*i *_that are annotated by both terms, A and B. With these assumptions, *P*(*A*, *B*) can be estimated, using the following equation:

$$P(A,B)=\frac{N(U_{1}^{A,B})+N(U_{2}^{A,B})}{N(U_{1})+N(U_{2})}$$

Similarly, we can estimate the value of *P*(*A*, $\bar{B}$) and *P*($\bar{A}$, *B*), and calculate the *Jaccard Similarity *between the term A and B. The output of the *Similarity Estimator *module is a *similarity matrix *of any pair of terms in the two taxonomies.

For quality consideration, those annotations without credible evidence, such as Inferred from Electronic Annotation (IEA), Non-traceable Author Statement (NAS), No biological Data available (ND), and Not Recorded (NR), are not be included. 512,721 annotations were used for the eventual construction of the *similarity matrix*.

To improve the match accuracy, the GLUE system uses a *Relaxation Labeler*, which searches for the match configuration that best satisfies the given domain constraints and heuristic knowledge. The key idea behind this approach is that the label of a node is typically influenced by *the features of the node's neighborhood *in the graph. For instance, if there are mappings between all children nodes of MF_*telomerase activity *_(GO:0003720) and CC_*telomerase catalytic core complex *_(GO:0000333), then the chance of "MF_*telomerase activity *_*mapping-to *CC_*telomerase catalytic core complex*_" will be increased. Two domain constraints were introduced into our project. One is that "If term A matches term B, then A also matches all parents of B" and the other is that "If all children of term A match term B then A also matches B". Based on the GLUE report, when the relaxation labeler was applied, the accuracy typically improved substantially in the first few iterations, and then gradually dropped. Because of this, we stopped the *Relaxation Labeler *operation after the first two iterations and generated a set of *match candidates*.

After validation, 15,232 one-to-one mappings were generated, covering almost half of all GO terms. As shown in Table 1, 3,882 (46%) terms of MF, 5,629 (39%) terms of BP and 1,233 (58%) terms of CC are involved in the mappings. Among them, 8401 (55%) paths focus on the relationships between Molecular Function and Biological Process (including MF2BP and BP2MF), while only 2014 (13%) paths start with Cellular Component (including CC2MF and CC2BP). The distribution of mapping types reflects the biased nature of current GO annotations.

**Table 1:** The distribution of one-to-one mappings.

**Mapping**	**Relation**	**Count**	**MF**	**BP**	**CC**
MF2CC	be-performed-in	2592	1397 (16%)		554 (33%)
CC2MF	performs	785	487 (6%)		347 (20%)
MF2BP	be-involved-in	5723	1822 (23%)	3327 (30%)	
BP2MF	involves	2678	1304 (17%)	1045 (10%)	
BP2CC	takes-on	2225		1019 (9%)	550 (33%)
CC2BP	undertakes	1229		1433 (13%)	372 (21%)
Total		15232	3882 (46%)	5629 (39%)	1233 (58%)

There are six types of mapping between the pairs of GO-terms coming from the three independent subontologies, covering almost half of all the GO terms.

### Inference rulebases

By introducing a set of inference rulebases, the *GORouter *will be able to provide semantic inference services. In addition to the two internal RDF and RDFS rulebases, the Oracle NDM also supports user-defined rulebases and uses them in specialized inferences across various RDF datasets.

**Figure 3:**
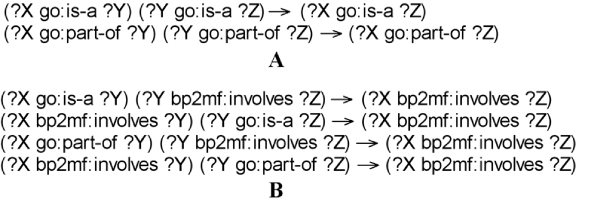
**User-defined: two types of inference rulebases of GORouter**. (A) The *True Path Rulebase *(RULE_GO) with two rules running on the GO subontologies dataset. (B) Each *Ontology Mapping Rulebase *(RULE_BP2MF) with four rules crossing three RDF dataset: BP, MF and BP2MF.

In this paper, we use two types of inference rulebases: *True Path Rulebase *(as shown in Figure 3A) and *Ontology Mapping Rulebases *(as shown in Figure 3B). The *True Path Rulebase *reflects the organization principle (i.e. "*true path rule*") within the *GO Subontology Datasets*. The *Ontology Mapping Rulebases *cover all permutations and combinations between *GO Subontology Datasets *and *Ontology Mapping Datasets*.

In the rulebases, each rule consists of three parts: an IF side pattern as the antecedents; an optional filter condition that further restricts the subgraphs matched by the IF side pattern; and a THEN side pattern for the consequents. To simplify the expression, we use the "→" character to separate the IF side pattern from the THEN side pattern, while optional filter conditions are omitted.

**Figure 4:**
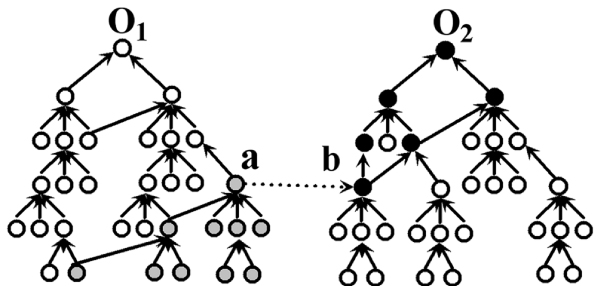
**An illustration of semantic inference running on the mapping directed from node a to b**. Given two ontologies *O*_1 _and *O*_2_, the sentence "a *mapping-to *b" (*a *∈ *O*_1_, *b *∈ *O*_2_) can be inferred by a reasoning engine. For any child node a_i _of a, we can infer that "a_i _*maps-to *b". Similarly, for any parent node b_j _of b, we can infer that "a *mapping-to *b_j_". Furthermore, for any child node a_i _of a, any parent node b_j _of b, the assertions of "a_i _*mapping-to *b_j_" are also valid.

Given two ontologies *O*_1 _and *O*_2 _(Figure 4), a sentence of the form "a *mapping-to *b" (where *a *∈ *O*_1_, *b *∈ *O*_2 _and "*mapping-to*" stands in for one of six mapping types) can thus be conceived as expressing general statements about the mapping between different GO subontologies. For any child node a_i _of a (the form "a_i _*is-child-of *a", where "*is-child-of*" stands for "*is-a*" or "*part-of*" expressions), we can infer that "a_i _*maps-to *b". Similarly, for any parent node b_j _of b (where "b *is-child-of *b_j_") we can infer that "a *mapping-to *b_j_". Furthermore, for any child node a_i _of a and any parent node b_j _of b, the assertion of "a_i _*mapping-to *b_j_" is also valid. By introducing inference rulebases, *GORouter *can infer a total of sixty results, which obey the same mapping from node a to node b.

### GORouter architecture

**Figure 5:**
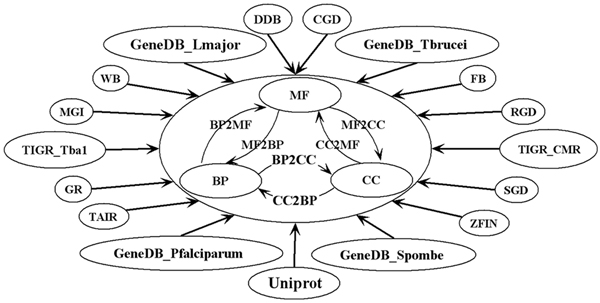
**The framework of GORouter**. *GORouter *organized with three-tier framework: 1) *Core Tier*: consists of 3 *GO Subontology Datasets *and 5 *GOSlim Datasets *(GOSlim_Generic, GOSlim_GOA, GOSlim_Plant, GOSlim_Prokaryotic and GOSlim_Yeast not display in this figure). 2) *Mapping Tier*: consists of the 6 *Ontology Mapping Datasets *generated by the GLUE system. 3) *Annotation Tier*: consists of 17 *GO Annotation Datasets*; filtering of the annotation files is provided by GO collaborating groups.

By integrating heterogeneous original data with uniform RDF format, creating additional mappings between pairs of terms coming from different GO subontologies, and introducing a set of reasoning rulebases across various RDF datasets, we produced the RDF model *GORouter *(As shown in Figure 5). In total, 31 RDF datasets and 7 RDF rulebases have been integrated into the *GORouter*.

Compared with the single term-centric XML-RDF data file, the RDF datasets are organized with a three-tier framework: 1) *Core Tier*: consists of 3 *GO Subontology Datasets *and 5 *GOSlim Datasets *(including GOSlim_Generic, GOSlim_GOA, GOSlim_Plant, GOSlim_Prokaryotic and GOSlim_Yeast). 2) *Mapping Tier*: consists of the 6 *Ontology Mapping Datasets *generated by the GLUE system. 3) *Annotation Tier*: consists of 17 *GO Annotation Datasets*; filtering of the annotation files is provided by GO collaborating groups.

Refining the set of mapping types simplifies the search statements. In the *GORouter*, we normalized the definition of relationships between the RDF datasets. Furthermore, when creating mappings, we used more restricted domain constraints. Hence, these mappings enrich the relationships and have the ability to provide complex semantic query and inference services.

## Application

A variety of applications that provide visualization and query capabilities for the GO are available. For example, the AmiGO , GoFish [39] and EP  browsers all use web interfaces to implement searching and displaying the ontology, term definitions and associated annotated gene products for the entire spectrum of contributing GO collaborating databases. Apart from the basic functions, however, there are profound differences between the various applications. For instance, GoFish provides Boolean queries of combinations of GO attributes, and the EP GO Browser provides clustering, analysis and visualization services. Unfortunately, although many applications use the GO subontologies or the gene associations, as well as similar development architectures, so far their integration has been problematic [40].

Stein et al., have suggested using two technologies, ontology and globally unique identifiers for the integration of biological databases. In constructing *GORouter*, we have followed this suggestion. We believe that this RDF model can partially overcome the problems described above, thus promoting information sharing and exchange among different research domains. Based on *GORouter*, we developed a prototype application to provide semantic query and inference services.

### Loading and tuning

In order to improve performance, we chose Oracle 10g NDM as the native RDF data repository and used table function RDF_MATCH [41] to seamlessly integrate multiple RDF datasets, RDF rulebases and traditional relational datasets into a rich SQL statement. As a result, the scale of *GORouter *is minimized and the speed of RDF retrieval is increased dramatically (as shown in Table 2). Data not involved in semantic inference are directly stored in Oracle relational tables. We believe that this is an effective way to partly overcome the bottleneck of conventional semantic web applications.

**Table 2:** The loading and querying performance analysis of three semantic web prototype applications.

**Application**	**Environment**	**Repository**	**Triples**	**Storage**	**Query Time**
GORouter	Dual processors of 1.66 GHz, 2 GB RAM	Oracle 10g NDM	5.5 M	Disk + Memory	0.74 s
AllegroGraph	Dual processors of 1.8 GHz, 16 GB RAM	AllegroGraph	6.88 M	Disk	172 s
YeastHub	Dual processors of 2 GHz, 2 GB RAM	Sesame	1.4 M	Memory	38 s

Performance analysis of three semantic web prototype applications:*GORouter, AllegroGraph and YeastHub*. In this table, the AllegroGraph  project used the disk approach to query "LUBM50, Lehigh U. Benchmark" datasets (about 6.88 million triples). The retrieval speed is about 0.04 million triples per second. The YeastHub  project uses the main memory approach to query the UniProt data file (about 1.4 million triples).

At present, the *GORouter *is about 210 MB (~5.5 million triple statements), including the essential annotations and their relationships. In comparison, the size of traditional relational data, such as GO term definition, gene product sequence, not creditable annotations, etc is over 4 GB. It took about 10 hours to convert and load these data into the Oracle database, most of which was spent in the initial loading of RDF datasets into the Oracle NDM repository.

We used a web server, running Red Hat Enterprise Linux AS release 3 (Taroon Update 2) with dual 1.66 GHz processors and 2 GB main memory. In order to attain better performance times, we created a set of indexes for RDF triples and in particular function-based indexes for RDF rulebases, adjusted the Java Virtual Memory heap size and Oracle SGA size, extended the size of temporary tablespace, and used the DBMS_STATS package to gather statistics about the physical storage characteristics of tables and indexes. As a result, the speed of semantic queries and inferences performed either on par with or slightly better than traditional relational queries.

### Examples of usage

Our example queries demonstrate how to use two types of inference rulebases to provide semantic query and inference services. In the following use cases, we attempt to show some improvement over the traditional GO query tools. To simplify RDF_MATCH search pattern across multiple RDF datasets, RDF rulebases and relational tables, we developed a set of APIs to translate user input from web form into rich SQL statement.

#### Case 1

**Figure 6:**
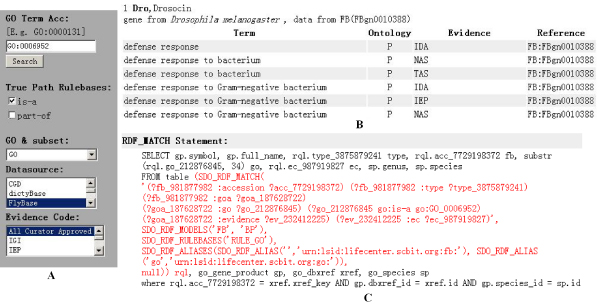
**Using True Path Rulebases to provide semantic inference services**. The screen shot consists of three components: (A) the query forms, (B) the partial output of example query, (C) the RDF_MATCH search pattern. Notice that, (C) is not shown on the GORouter website. This use case applies *True Path Rulebases *(GLUE_GO) to replace traditional 'graph_path' table of AmiGO to provide reasoning services of transitive correlations.

This use case applies *True Path Rulebases *to replace traditional 'graph_path' table of AmiGO to provide reasoning services of transitive correlations. Figure 6 shows a query form that fetch annotations for fly gene products associated to BP_*defense response *_(GO: 0006952) or any of its children with '*is-a*' relationship. We believe this solution provides greater flexibility for users. For example, we can remove rulebases from query statement to see direct correlations of GO-terms. Furthermore, we can use certain *GOSlim Dataset *to replace *GO Subontology Dataset *to limit the scope of query.

#### Case 2

**Figure 7:**
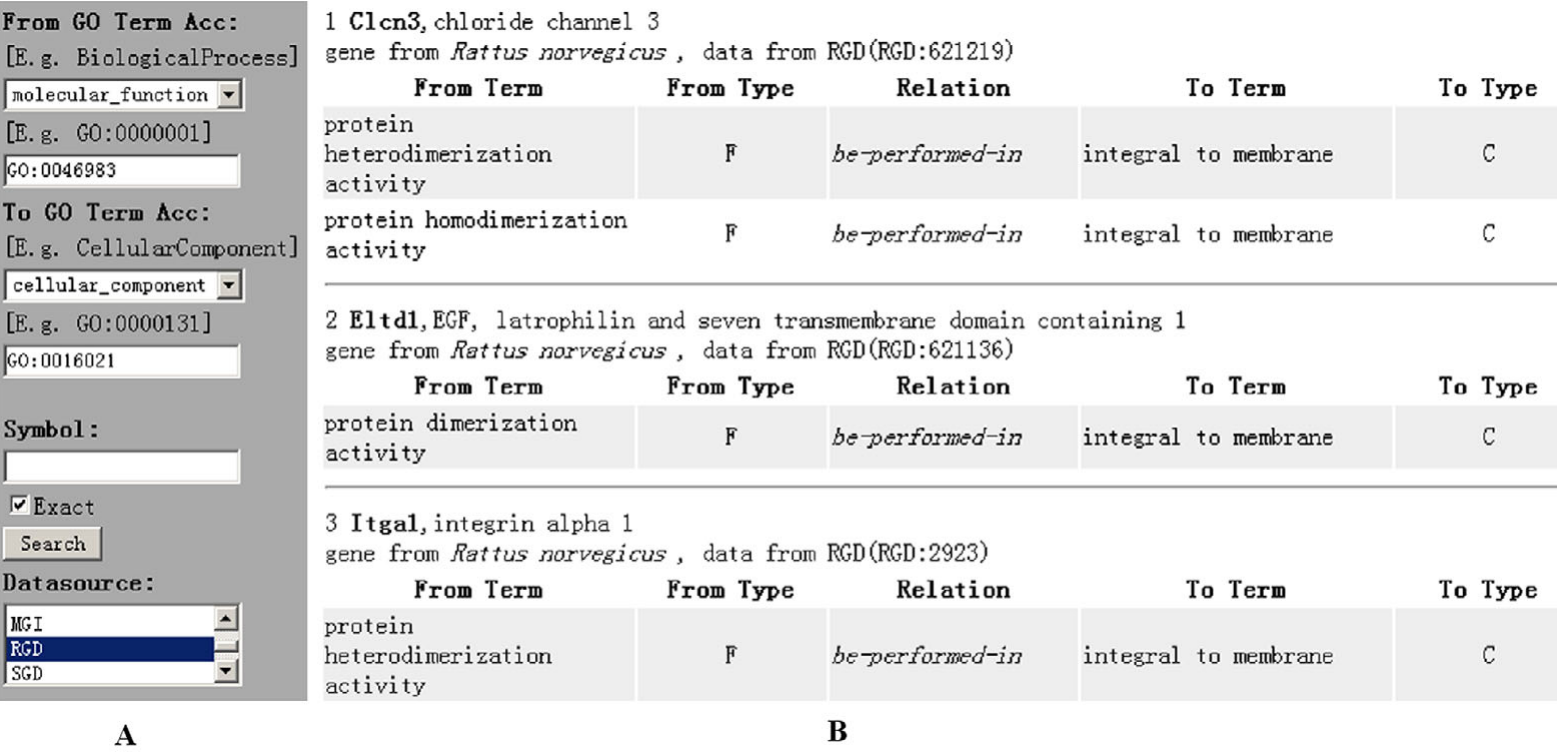
**Using Ontology Mapping Rulebases to provide semantic inference services**. The screen shot consists of two components: (A) the query forms, (B) the partial output of example query. This use case across three *RDF datasets *(MF, CC, and MF2CC) and one *Ontology Mapping Rulebase *(RULE_MF2CC), that fetch gene products of Rat Genome Database (RGD) associated with MF_*protein dimerization activity *_(GO:0046983) and CC_*integral to membrane *_(GO:0016021).

This use case applies *Ontology Mapping Rulebase *to provide inference service across various *GO Subontology Datasets*. In the study of *rattus norvegicus*, we are interested to find out what type of dimerization activity is taken place. Figure 7 shows a query form, crossing three *RDF datasets *(MF, CC, and MF2CC) and one *Ontology Mapping Rulebase *(RULE_MF2CC), that fetch gene products of Rat Genome Database (RGD) associated with MF_*protein dimerization activity *_(GO:0046983) and CC_*integral to membrane *_(GO:0016021). The result shows that the interactions between the gene products, Clcn3, could involve an association between identical proteins (homomers) or non-identical proteins (heteromers). As we know, both MF_*protein heterodimerization activity *_(GO:0046982) and MF_*protein homodimerization activity *_(GO:0042803) are belonging to MF_*protein dimerization activity*_. The inference could be beneficial to the experiment design for future researches. In contrast, through the same query we also find some other gene products, for example, Eltd1, which performs only protein dimerization activity and can be retrieved by the traditional tools.

## List of abbreviations used

MF – Molecular Function Subontology.

BP – Biological Process Subontology.

CC – Cellular Component Subontology.

MF2BP – The mapping dataset directed from MF to BP which relation is "*be-involved-in*".

BP2MF – The mapping dataset directed from BP to MF which relation with "*involves*".

MF2CC – The mapping dataset directed from MF to CC which relation is "*be-performed-in*".

CC2MF – The mapping dataset directed from CC to MF which relation is "*performs*".

BP2CC – The mapping dataset directed from BP to CC which relation is "*takes-on*".

CC2BP – The mapping dataset directed from CC to BP which relation is "*undertakes*".

## Competing interests

The authors declare that they have no competing interests.

## Authors' contributions

YL and QL generated the original idea, QX executed the research, QX and YS wrote the paper. GZ participated in the design of the model. QL conceived of the study, and participated in its design and coordination and helped to draft the manuscript. All authors have read and approved the final manuscript.
